# Multimorbidity Patterns in the General Population: Results from the EpiChron Cohort Study

**DOI:** 10.3390/ijerph17124242

**Published:** 2020-06-14

**Authors:** Ignatios Ioakeim-Skoufa, Beatriz Poblador-Plou, Jonás Carmona-Pírez, Jesús Díez-Manglano, Rokas Navickas, Luis Andrés Gimeno-Feliu, Francisca González-Rubio, Elena Jureviciene, Laimis Dambrauskas, Alexandra Prados-Torres, Antonio Gimeno-Miguel

**Affiliations:** 1 EpiChron Research Group, IIS Aragón, 50009 Zaragoza, Spain; ignacio.ioakim@hotmail.es; 2 EpiChron Research Group, Aragon Health Sciences Institute (IACS), IIS Aragón, Miguel Servet University Hospital, 50009 Zaragoza, Spain; bpoblador.iacs@aragon.es (B.P.-P.); jcarmona@iisaragon.es (J.C.-P.); jdiez@aragon.es (J.D.-M.); lugifel@gmail.com (L.A.G.-F.); franciscagonzalezrubio@gmail.com (F.G.-R.); sprados.iacs@aragon.es (A.P.-T.); 3 Health Services Research on Chronic Patients Network (REDISSEC), ISCIII, 28222 Madrid, Spain; 4 Aragon Health Service (SALUD), Delicias-Sur Primary Care Health Centre, 50003 Zaragoza, Spain; 5 Aragon Health Service (SALUD), Internal Medicine Service, Royo Villanova Hospital, 50015 Zaragoza, Spain; 6 Faculty of Medicine, Vilnius University, LT-03101 Vilnius, Lithuania; rokas.navickas@santa.lt (R.N.); Elena.Jureviciene@santa.lt (E.J.); Laimis.Dambrauskas@santa.lt (L.D.); 7 Department of Biomedical Research, Vilnius University Hospital Santaros Klinikos, LT-08661 Vilnius, Lithuania; 8 Aragon Health Service (SALUD), San Pablo Primary Care Health Centre, 50003 Zaragoza, Spain; 9 Department of Medicine, University of Zaragoza, 50009 Zaragoza, Spain; 10 Grupo de Trabajo de Utilización de Fármacos de la SemFYC, 28004 Madrid, Spain

**Keywords:** age, chronic diseases, factor analysis, multimorbidity, patterns, real-world data, sex, Spain

## Abstract

The correct management of patients with multimorbidity remains one of the main challenges for healthcare systems worldwide. In this study, we analyze the existence of multimorbidity patterns in the general population based on gender and age. We conducted a cross-sectional study of individuals of all ages from the EpiChron Cohort, Spain (1,253,292 subjects), and analyzed the presence of systematic associations among chronic disease diagnoses using exploratory factor analysis. We identified and clinically described a total of 14 different multimorbidity patterns (12 in women and 12 in men), with some relevant differences in the functions of age and gender. The number and complexity of the patterns was shown to increase with age in both genders. We identified associations of circulatory diseases with respiratory disorders, chronic musculoskeletal diseases with depression and anxiety, and a very consistent pattern of conditions whose co-occurrence is known as metabolic syndrome (hypertension, diabetes, obesity, and dyslipidaemia), among others. Our results demonstrate the potential of using real-world data to conduct large-scale epidemiological studies to assess the complex interactions among chronic conditions. This could be useful in designing clinical interventions for patients with multimorbidity, as well as recommendations for healthcare professionals on how to handle these types of patients in clinical practice.

## 1. Introduction

Multimorbidity (i.e., the presence of more than one chronic disease in an individual) is a common finding in the general population, particularly in older adults, posing a big challenge for patients and health systems worldwide due to its great impact on health and healthcare [1,2]. To face this problem, some initiatives such as Joint Actions Chrodis [3] and Chrodis-Plus [4] have been developed at a European level to develop and pilot the implementation of integrated care models for multimorbidity in different care settings. Other care models for tackling multimorbidity, like the Ariadne principles [5], have been designed to be incorporated into family physicians’ daily practice, and also into educational programs for health professionals on the care of patients with multimorbidity.

Prior to the development and implementation in clinical practice of this kind of models, we need to generate evidence on the epidemiology of multimorbidity and its impact on health through clinical trials and observational studies. Several clinical trials and interventions have been conducted in recent years with different specific aims and targeting specific sectors of the population with multimorbidity and polypharmacy. To name just a few, we can highlight the Prioritizing Multimedication in Multimorbidity (PRIMUM) Study in primary care [6], the Multimorbidity and Polypharmacy in Primary Care (MULTIPAP) Study [7], the 3D Study [8], the Supporting prescribing in older patients with multimorbidity and significant polypharmacy (SPPiRE) trial [9], the six-week occupation-based self-management OPTIMAL program [10], or the Health Teams Advancing Patient Experience: Strengthening Quality (TAPESTRY) intervention [11], among others.

Nonetheless, clinical trials might present some limitations related to the comparability and external validity of results due to the limited number of subjects recruited or to the inclusion and exclusion criteria applied, and to their high economic cost associated. On the other hand, the increasing availability for health research of real-world data generated during the care process, such as electronic health records (EHRs), represents a cheaper opportunity to generate real-world evidence about the epidemiology of multimorbidity through population-based observational studies.

In this context, the EpiChron Cohort Study was initiated in 2011 as a large-scale population-based study on the epidemiology of chronic diseases and multimorbidity through the use of real-world data in the Spanish region of Aragón [12]. One of the specific objectives of this project was to study the existence of systematic associations among chronic diseases. Understanding how the diseases tend to cluster in the form of multimorbidity patterns could serve to obtain useful information on common underlying pathophysiological mechanisms and to generate new hypotheses (e.g., clinical and pharmacological). This could help us to develop new clinical guidelines adapted for people with multiple chronic diseases, based on the most relevant and frequent associations found among diseases. This type of analysis has already been conducted in the bibliography and also by our research group in the adult population and in specific subpopulations using similar data [13,14,15,16,17,18,19,20], but not in the whole of the general population, including information on all chronic diagnoses from both primary and hospital care. The effects of gender and age on the composition of multimorbidity patterns have not been exhaustively analyzed either.

During a study of associations among diseases, it is crucial to focus on the role of sex and age. The distribution of diseases differs between women and men throughout their different stages of life. Moreover, the European Commission promotes an inclusive perspective of sex and gender in medical research funded by the European Union [21]. On the other hand, if we can identify how and when diseases tend to add to a given pattern, we could anticipate and direct primary prevention strategies to younger population groups to prevent or delay the development of specific conditions or disease combinations.

This large-scale population-based study aimed to exhaustively analyze the existence of systematic associations among chronic diseases in the general population based on sex and age, and to describe and clinically discuss the patterns of multimorbidity identified.

## 2. Materials and Methods 

### 2.1. Design and Study Population

We conducted a retrospective, observational study based on data from the EpiChron Cohort Study. This cohort links demographic and clinical information for all public health system users of the Spanish region of Aragón (approximately 1.3 million inhabitants). A description of the cohort profile, of the original data sources used, and of data curation procedures was published elsewhere [12]. We selected, for this study, all individuals with at least one contact with the public health system registered in their EHRs between 1 January 2011 and 31 December 2011 (i.e., 1,253,292 patients). We stratified individuals by sex and into seven age groups: 0–14, 15–29, 30–44, 45–59, 60–74, 75–89, and ≥90 years.

### 2.2. Variables

For each participant, we analyzed sex, age, and all chronic conditions present during the year of study. Diagnoses were extracted from the EHRs of both primary and hospital care levels, and included all active episodes between 1 January 2011 and 31 December 2011, even if a clinician had recorded the diagnosis before the initial date. Diseases were coded following the International Classification of Primary Care (ICPC) or the International Classification of Diseases, Ninth Revision, Clinical Modification (ICD-9-CM), and then grouped into 260 mutually exclusive Expanded Diagnostic Clusters (EDCs) using the Johns Hopkins Adjusted Clinical Groups (ACG®) System (version 11.0, The Johns Hopkins University, Baltimore, MD, USA) [22]. We considered for the analysis all 114 possible EDCs previously defined as chronic by Salisbury et al. [2]. We defined multimorbidity as the presence of more than one EDC from Salisbury’s list.

The Clinical Research Ethics Committee of Aragón (CEICA) approved this study (Research protocol PI17/0024). The CEICA waived the requirement to obtain informed consent from patients since all the information used was anonymized.

### 2.3. Statistical Analysis

#### 2.3.1. Descriptive Analysis

We performed a descriptive analysis of the demographic (i.e., sex and age) and clinical (i.e., number of chronic diseases and proportion of multimorbidity) characteristics of the study population. The results were expressed as means accompanied by their respective standard deviations or as absolute and relative frequencies and proportions accompanied by their respective 95% confidence intervals (CI).

#### 2.3.2. Factor Analysis 

We applied exploratory factor analysis to each sex and age group to identify the existence of systematic associations among chronic diseases. Exploratory factor analysis is a statistical method aimed at uncovering the underlying structure of a large set of variables (e.g., diseases). In our study, factor analysis was used to identify sets of chronic diseases with a common underlying causal factor (i.e., factors or patterns). To facilitate the clinical–epidemiological interpretation of the results, we only included in the factor analysis those chronic comorbidities with prevalence equal to or greater than 1% in each sub-population.

The methodology followed was described elsewhere [13]. We coded EDCs as binary variables based on the presence/absence of each condition (i.e., zero = no disease; one = presence of the disease). We applied a factor analysis based on a correlation matrix to determine which conditions comprised each factor. Due to the dichotomous nature of the variables, we used tetrachoric correlation matrices. We used the principal factor method for the extraction of the factors, and a scree plot of the eigenvalues of the correlation matrix represented in descending order, in combination with clinical criteria, to decide the number of factors to extract. We applied an oblique rotation (Oblimin) to facilitate the interpretation of the factors. Each disease within a factor had a factor score with a value between −1 and one, representing the association of each of the diagnoses with its disease pattern. We considered those EDCs with factor scores ≥0.30 within a pattern to be part of that pattern and allowed the same EDC to be present in various patterns. Those EDCs with factor scores ≥0.25 and <0.30 were also included in a pattern if helped with the clinical interpretation of such pattern. We analyzed model goodness-of-fit and sample adequacy using the Kaiser–Meyer–Olkin (KMO) parameter, which takes values between zero (lower) and one (greater goodness of fit).

We conducted all statistical analyses in STATA (Version 12.0, StataCorp LLC, College Station, TX, USA), with statistical significance set at *p* < 0.05.

### 2.4. Name and Clinical Consistency of the Patterns

In order to estimate the prevalence in the population of the resulting multimorbidity patterns, we assigned individuals to a given pattern if they had at least two of the diseases that comprised the pattern. After performing factor analysis, seven clinicians specialized in Internal Medicine, Cardiology, Family and Community Medicine and Preventive Medicine and Public Health (I.I.S., J.C.P., J.D.M., L.A.G.F., F.G.R., R.N. and A.P.T.) proposed a name for each of the identified disease combinations to facilitate the interpretation of the results and increase the interest of the study. The denomination of the patterns was agreed according to the most relevant diseases within each pattern, considering their factor score, prevalence and clinical relevance, and the denominations already used in the literature. In most of the cases, the denomination was based on the main organ systems affected. Particularly in the case of the endocrine system, when obesity, diabetes, dyslipidemia, and hypertension were associated in a single pattern, we described this concrete pattern as metabolic, as it is composed of conditions whose coexistence could potentially lead to the diagnosis of metabolic syndrome if certain criteria are met.

## 3. Results

### 3.1. Overall Characteristics of the Study Population

The sex and age distributions of the study population (50.5% women; mean age of 44.2 years), together with the mean number of chronic diseases and the prevalence of multimorbidity, are shown in Table 1. Women presented, on average, 0.5 more diseases than men, and the morbidity burden increased with age from 0.57 diseases in subjects aged 0–14 years to 4.35 diseases in those aged 75–89 years, and then decreased to 3.35 diseases in elders aged ≥90 years. Similarly, women showed a 10% higher prevalence of multimorbidity than men, which followed a similar trend to that of the mean number of diseases, increasing from 12.0% in children to 84.3% in 75–89-year-olds, and then decreasing to 68.3% in the oldest old.

**Figure 1:**
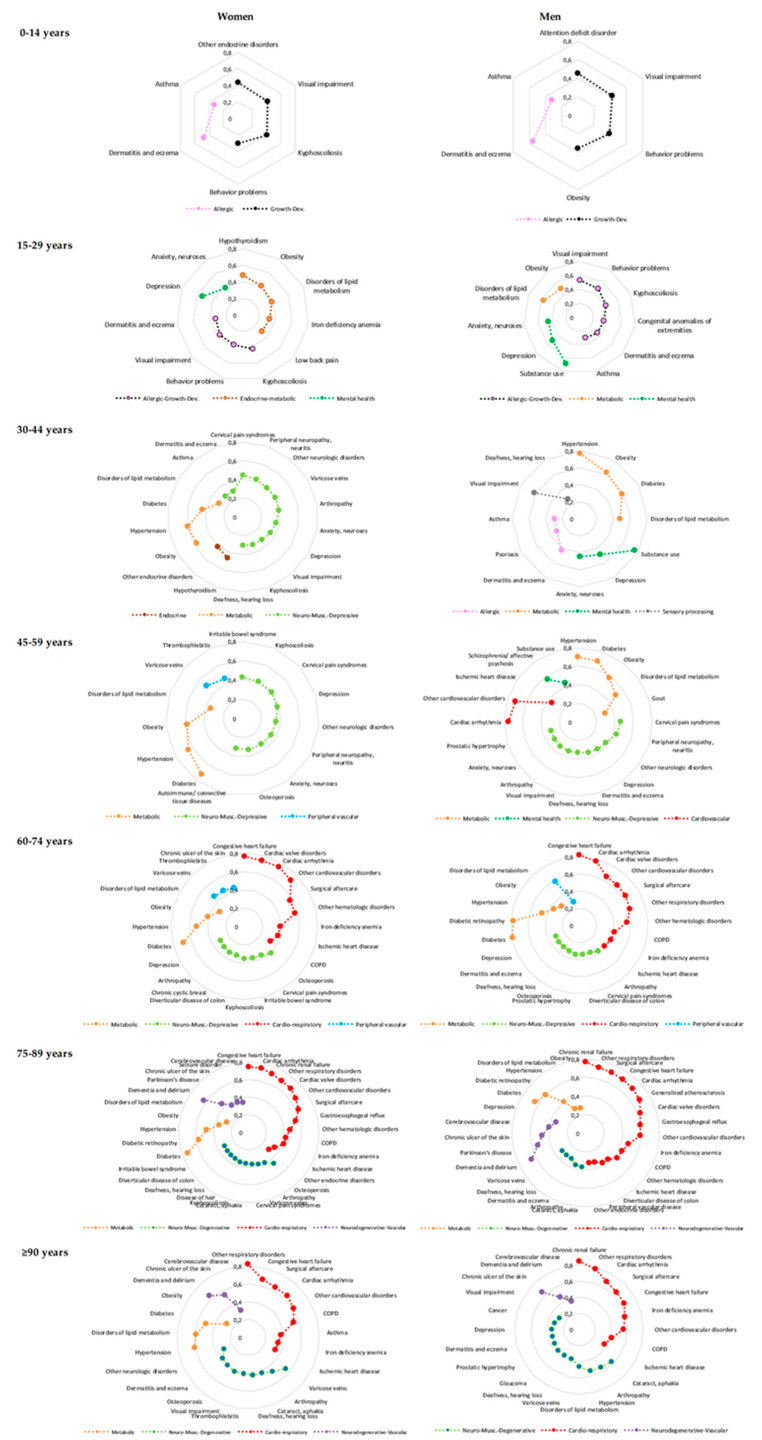
Disease composition of the multimorbidity patterns identified in the study population based on sex and age.

We identified a total of 14 different multimorbidity patterns; 12 in women, and 12 in men (Table 2). In each sex, we found two patterns in the pediatric population and three to four in the older age groups. On average, the number of diseases per pattern increased from three in the pediatric population to seven in patients 75 years of age and older. The most common pathologic conditions presented in the identified patterns included diseases of the circulatory system (ischemic heart disease, cardiac arrhythmia, congestive heart failure), respiratory disorders (asthma, emphysema, chronic bronchitis, chronic obstructive pulmonary disease), diseases of the musculoskeletal system (arthropathy, osteoporosis, cervical pain syndromes), mental and behavioral disorders (depression, anxiety and neuroses, substance use disorder, schizophrenia), and endocrine diseases (diabetes, obesity, hypothyroidism) (Figure 1).

The complete output of the factor analysis is presented in the Supplementary Material (Tables S1 and S2), in which we included the scree plots used to decide the numbers of patterns to be extracted in each group, the proportion of cumulative variance explained, the KMO parameter in each sub-analysis, and the complete list of diseases analyzed in each age group together with their prevalence, EDC code, and factor score (even if they were not part of any pattern).

### 3.2. Multimorbidity Patterns in Women

In the pediatric population, two in 100 individuals presented an allergic pattern consisting of dermatitis, eczema, and asthma. In adolescents and young adults between 15–29 years of age, these conditions were associated with growth and developmental disorders. Depression, anxiety and neurosis were associated in a mental health pattern in two in every 1000 women between 15–29 years old. In older age groups, up to 74 years of age, depression was associated with musculoskeletal conditions.

We identified systematic associations of risk factors for heart disease, mainly diabetes and hypertension, in the female population 30 years of age and older. This metabolic pattern was the most prevalent in women between 45–74 years old. One in three women aged 60–89 years presented a metabolic pattern, whereas in older women this proportion decreased to one in five. In younger women aged 15–29 years, an endocrine–metabolic pattern included hypothyroidism, obesity and dyslipidemia among the primary disorders. We also identified an endocrine pattern in women between 30–44 years old, with hypothyroidism and other endocrinologic diseases. 

Many diseases of the circulatory system were systematically associated in women aged 60 years and older. The leading chronic conditions were congestive heart failure, cardiac valve disorders, and cardiac arrhythmia. Various diseases of the respiratory system were also included in this cardiorespiratory pattern, especially among women 75 years of age and older. We also identified a peripheral vascular pattern in the age range of 45–74 years, in which varicose veins of lower extremities and thrombophlebitis were the main conditions. In older women, dementia and delirium, Parkinson’s disease, and chronic ulcers of the skin were the primary disorders of a neurodegenerative–vascular pattern.

Diseases of the musculoskeletal system were systematically associated with a variety of chronic conditions in the different age groups. In the pediatric population, we identified a growth–developmental pattern, with kyphoscoliosis, behavior problems and endocrine disorders. In women between 15–29 years old, kyphoscoliosis was associated with behavior problems and dermatitis and eczema, in an allergic–growth–developmental pattern. In women between 30–74 years of age, we identified a neuromusculoskeletal–depressive pattern, with kyphoscoliosis, cervical pain syndromes, peripheral neuropathy, neuritis and depression among the main pathologic conditions. Osteoporosis was also among the leading conditions of this pattern, especially in women 60–74 years of age. In older age groups, a neuromusculoskeletal–depressive pattern included arthropathy, osteoporosis, varicose veins of the lower extremities, cataracts, aphakia, and hearing loss; this pattern was the most prevalent in women over 75 years of age.

### 3.3. Multimorbidity Patterns in Men

We identified an allergic pattern in the pediatric population and men between 30–44 years of age. This pattern included dermatitis, eczema, and asthma; in the age group 30–44 years old, it also included psoriasis. In adolescents and young adults aged 15–29 years, allergic conditions were associated with growth and developmental disorders.

In men, a metabolic pattern was the most common pattern in every age group in which it was present—from 15 to 89 years old. In the pediatric population, obesity, behavior problems, and attention deficit disorder were among the main conditions of a growth–developmental pattern. 

We observed systematic associations with various chronic diseases of the circulatory system above 45 years of age. A cardiovascular pattern in men between 45–59 years old included cardiac arrhythmia, ischemic heart disease, and other cardiovascular disorders; its prevalence was approximately four in every 1000 men. In men over 60 years of age, congestive heart failure and other circulatory diseases were associated with emphysema, chronic bronchitis, chronic obstructive pulmonary disease, and other respiratory disorders in a cardiorespiratory pattern. 

In men 60–74 years old, we found a peripheral vascular pattern with varicose veins of lower extremities and chronic ulcer of the skin. In older men, we observed a neurodegenerative–vascular pattern. 

Depression was associated with many other chronic pathologic conditions in men above 15 years of age, except in the group aged 75–89 years old. We identified a mental health pattern in men 15–59 years of age. Substance use disorder was associated with depression, anxiety and neurosis in men 15–44 years old, and with schizophrenia and affective psychosis in men 45–59 years old. In older age groups, depression was associated with various diseases of the musculoskeletal system.

A pattern of vision problems and hearing loss was described in men aged 30–44 years, otherwise referred to as a sensory processing pattern. These conditions were associated with musculoskeletal diseases in other age groups, vision problems in individuals from 15 to 59 years of age, and hearing loss in those over the age of 30 years. In the pediatric population, vision problems were included in the growth–developmental pattern.

As observed in women, diseases of the musculoskeletal system were also systematically associated with a variety of different chronic conditions in men. In adolescents and young men between 15–29 years old, kyphoscoliosis and congenital anomalies of the extremities were associated with behavior problems, asthma, and dermatitis and eczema in an allergic–growth–developmental pattern. In men between 45–74 years old, we found a neuromusculoskeletal–depressive pattern with cervical pain syndromes, depression, and peripheral neuropathy among the primary pathologic conditions. In men over 60 years of age, arthropathy was also among the leading conditions of this pattern. In men above 75 years of age, arthropathy was associated with ophthalmologic conditions such as cataracts and aphakia, varicose veins of the lower extremities, and various other chronic conditions, in a neuromusculoskeletal–degenerative pattern. This was the most common multimorbidity pattern in men above 90 years of age, being present in approximately one in two individuals.

## 4. Discussion

This population-based study shows that multimorbidity affects people of all ages, and thus that it should not be seen as a simple problem of ageing. Even in the pediatric population, we found that one in ten individuals had at least two chronic diseases. We were also able to identify systematic associations between various chronic conditions in all age groups and in both genders. We saw that multimorbidity tends to cluster diseases not necessarily from the same specialty; this observation constitutes a challenge for clinicians who often need to stretch their competencies, as well as for patients who may be advised to combine two or more slightly different guidance proposals. 

Multimorbidity constitutes a severe problem for healthcare systems, as it is associated with poorer health outcomes and more frequent use of healthcare services, as well as higher costs [23,24]. It has been estimated that about 50 million people in the European Union live with multiple chronic diseases [25,26]. Although a significant part of healthcare resources support patients with multimorbidity [26], we have not achieved considerably improved health outcomes in this population. There are several widely used risk assessment tools for some chronic conditions, such as the Framingham risk score and the Systematic Coronary Risk Evaluation (SCORE) risk charts for cardiovascular diseases [27,28]. However, the presence of complex conditions in patients with multimorbidity is more challenging to assess. The prevention of either the deterioration or co-occurrence of additional comorbidity is still a challenge for clinicians, partially due to a lack of specific assessment tools. Therefore, there is a lot of interest in designing new approaches for patients with multiple chronic conditions. In order to achieve that, more work on the epidemiology of multimorbidity is needed, based especially on large, less biased, real-world data. 

Studies focused on multimorbidity patterns may provide useful information on how to improve preventive strategies and clinical management in patients with multiple chronic conditions. The identification of systematic associations between chronic diseases beyond chance is essential, in order to generate new hypotheses about biological interactions among diseases, and to study the impact of the morbidity burden on health outcomes, from public health and person-centered perspectives [29,30]. 

There is a great diversity in the medical literature regarding the nature and prevalence of multimorbidity patterns across different studies, partially due to different methodological approaches used on selecting and coding diseases, as well as due to different population samples [31]. A more uniform methodological framework would improve the external validity and generalizability of the findings, and this is crucial mainly due to the uncertainty about the effectiveness of interventions that are targeted at people with multiple chronic conditions [32,33]. In this study, we aimed to provide a detailed description of the methodology used, so that our readers could correctly interpret our results and make plausible comparisons with findings from other studies. We performed a factor analysis using tetrachoric correlation matrices, as this is the most widely used analytical approach for identifying multimorbidity patterns [34]. Moreover, we included all chronic conditions that were registered in the EHRs of the patients, in order to enhance external validity [33,34].

One of the most consistent patterns among the different age groups, and in both genders, was the metabolic one, consisting of hypertension, diabetes, dyslipidemia and obesity. Patients with a metabolic pattern may present a metabolic syndrome, if certain criteria are met [35,36,37]. In men 45–59 years old, gout was also associated with a metabolic pattern. The association between gout and metabolic pattern is well described in the literature [38], although, in some studies, gout was observed in older age groups [13,15]. The associations between gout and metabolic syndrome could be partially explained as a result of causal comorbidity, due to the common risk factors that these conditions share [39,40,41]. Although the metabolic pattern was common among old adults, it seems that it may also be present in younger age groups. Previous studies reported a cardiometabolic pattern, which included a combination of metabolic pattern conditions with cardiovascular diseases, such as cardiac arrhythmia and angina, especially among the elderly [13,15,38,41]. In this cardiometabolic pattern, cardiovascular diseases such as ischemic heart disease, congestive heart failure, and cardiac arrhythmia, were usually observed after the age of 45 years old and even later in life in women [13,15]. In our study, such cardiovascular diseases were not associated with metabolic syndrome, but with respiratory disorders. Our findings suggest that a cardiovascular pattern can be observed earlier in life in men than in women. With time, this pattern also appears to include respiratory disorders, such as obstructive pulmonary disease. A cardiorespiratory pattern has also been reported by other authors, especially in studies where cardiovascular and respiratory diseases were not associated with metabolic syndrome [42,43,44]. Chronic obstructive pulmonary disease often coexists with various cardiovascular diseases, such as ischemic heart disease, heart failure and cardiac arrhythmia [45,46,47]. These conditions share common risk factors, such as smoking and systemic inflammation, and their coexistence is associated with poorer health outcomes [45,46,47]. It has been reported that patients with chronic obstructive pulmonary disease, especially in the under 65s (mid to late middle age), have an increased risk of cardiovascular comorbidity [45].

Among the most intricate patterns in this study, due to the wide variety of conditions they included, were the systematic associations between musculoskeletal disorders and other chronic diseases. Similar patterns have also been reported in the literature, with different names used by the authors to describe them [38,41]. In the pediatric population and young individuals up to 29 years old, kyphoscoliosis was associated with visual impairment and behavior problems. In adults, especially in those patterns that musculoskeletal diseases could cause chronic algic conditions, such as arthropathy and cervical pain syndromes, mental health comorbidity was frequently present. The most common mental health conditions associated with musculoskeletal and neurologic diseases in our study were depression, anxiety and neuroses. The association between depression and anxiety is well documented in the literature, as also is the association between depression and neurological disorders [48,49]. Various studies identified a pattern with low back pain, arthropathy and/or osteoporosis associated with either one or both mental health diseases [15,19,30,38]. An association between chronic pain and depression has also been reported in the literature [50,51,52], but more research is needed in this field, mainly to identify possible common neurophysiological coactivating factors, including medications [53]. Depression and chronic inflammation are both linked to numerous pathologic conditions [54,55,56,57]. In men 75–89 years old, we found that depression was associated with neurodegenerative diseases. Depression and anxiety are common comorbidities in patients with neurodegenerative conditions, such as Alzheimer’s disease and Parkinson´s disease [58,59]; however, more studies are needed to understand how these conditions interact. The relationship between depression and dementia is fascinating and many researchers have tried to reveal its nature [60]. 

Both factor analysis and hierarchical clustering studies have identified a pattern of mental health problems, mainly consisting of depression and anxiety [15,19,34]. Some studies have reported that migraines may also be included in this pattern [34]. Although few authors include substance use disorders in their study, there are reports of systematic associations between these conditions and depression in men [13,15]. Numerous studies found strong associations between substance use disorders and mental health conditions as well, such as depression and psychosis [61,62,63,64]. Another important issue is that substance use disorders include substances that could establish a potential risk of drug–drug interactions with psychotropic medication, as is the case of alcohol, which could result in sedation and drowsiness [19,65]. A recent study reported important cases of drug–drug interactions and prescribing cascades in many multimorbidity patterns, especially in patterns with mental health problems and respiratory conditions [19]. 

One of the main priorities for public health systems worldwide is to improve healthcare practices for patients with multiple chronic conditions by implementing a person-centered approach. The prevention or proactive follow-up of complex multimorbid patients may have a better chance if it is based on information extracted by broad national population-level data analysis. Pattern analysis or clustering could provide a method through which to design educational activities for healthcare professionals on how to handle patients with multiple chronic conditions and to use the available resources efficiently. Besides the positive outcomes of analyzing multimorbidity patterns, it is too early to know if these could or should be used when predicting the progress or complexity of the patient or disease. Future longitudinal studies might provide more information on these aspects, especially to identify possible common risk factors for multimorbidity in the general population and specific risk factors for additional comorbidity in patients who already present a multimorbidity pattern.

The specifics of this work, which are crucial when counting on it for support in decision making, are that it is based on a large-scale population study, including 98% of the reference population. Moreover, we exhaustively studied the multimorbidity of the population by including in the analyses a total of 114 chronic condition diagnoses that were extracted from patients´ EHRs from both primary and hospital care, and not only the most prevalent or severe diseases, commonly limited to 40 chronic conditions, while also grouping some, which might have affected the findings. Counting on the data from patients´ EHRs and expanding it to primary and hospital care allows us to have a less biased perspective, compared to single-source or patient-reported data analysis. In addition, data in the EpiChron Cohort undergoes continuous quality control checkups that ensure its accuracy and reliability for research purposes, despite the fact that EHRs were not originally created for research. Moreover, we imposed no age restrictions on our study of multimorbidity patterns, compared to most studies that focus only on the oldest of the old or on populations over 18 years of age at most.

One of the main limitations of this work lies in the cross-sectional nature of the study. We characterized the multimorbidity patterns for a given timeframe, without considering either the time elapsed since the diagnosis of each disease, or the chronological appearance of comorbidities over time. In this regard, we aimed to study the baseline multimorbidity patterns of the population at entry to the cohort, and our next step will be to conduct longitudinal studies to analyze the evolution of the patterns over a 10-year period. Another limitation of our study is the unavailability of certain variables that would have been of interest for studying the differences in the multimorbidity patterns based on sociodemographic factors, lifestyle, clinical and functional variables.

## 5. Conclusions

Multimorbidity is one of the big challenges the ageing population is facing, while healthcare is unprepared to treat or oversee many conditions all at once, with very high quality but extremely specialized medical personnel on the battleground. Large population-level databases are becoming available and, with increasing data mining capabilities as well as artificial intelligence, it is time to start benefiting from real-world data when planning healthcare resources and educational programs for both healthcare professionals and patients. Clinical–epidemiological studies like ours are needed to reveal systematic associations between chronic conditions in the form of disease patterns that share similarities and differences between genders and age groups. Our findings may provide useful information in designing models of care for patients with multiple chronic conditions, encouraging proactiveness, and supporting clinicians with useful and appropriate tools.

## Figures and Tables

**Table 1:** Characteristics of the study population.

Population	N (% ^a^)	Mean Number of Diseases ^b^ (SD ^c^)	% ^e^ of Multimorbidity ^d^ (95% CI)
Age (years)			
0–14	150,331 (12.0)	0.57 (0.81)	12.0 (11.9–12.2)
15–29	191,109 (15.2)	0.53 (0.86)	11.6 (11.5–11.8)
30–44	314,977 (25.1)	0.76 (1.12)	18.8 (18.7–19.0)
45–59	262,804 (21.0)	1.60 (1.77)	41.9 (41.8–42.1)
60–74	180,137 (14.4)	3.23 (2.47)	73.7 (73.5–73.9)
75–89	137,694 (11.0)	4.35 (2.95)	84.3 (84.1–84.5)
≥90	16,240 (1.3)	3.35 (2.98)	68.3 (67.6–69.0)
Sex			
Men	620,661 (49.5)	1.41 (2.04)	32.2 (32.1–32.4)
Women	632,631 (50.5)	1.90 (2.32)	42.6 (42.5–42.8)
Total	1,253,292 (100)	1.66 (2.20)	37.5 (37.4–37.6)

^a^ These percentages refer to the total population; ^b^ from a list of 114 chronic conditions; ^c^ standard deviation; ^d^ defined as two or more diseases from the list of 114 chronic conditions; ^e^ these percentages refer to each age subpopulation.

**Table 2:** Number, denomination, prevalence, and number of diseases of the multimorbidity patterns found in the study population based on sex and age.

	Age (Years)						
**Women**	**0–14**	**15–29**	**30–44**	**45–59**	**60–74**	**75–89**	**≥90**
Number	2	3	3	3	4	4	4
Name (prevalence; No. of dis. ^a^)	Growth–dev. ^b^ (1.0%; 4 dis.)	Allergic Growth–dev. (1.2%; 4 dis.)	Endocrine (0.3%; 2 dis.)	Peripheral vascular (2.5%; 3 dis.)	Peripheral vascular (6.8%; 4 dis.)	Neurodegenerative–vascular (4.0%; 5 dis.)	Neurodegenerative–vascular (6.9%; 3 dis.)
	Allergic (2.0%; 2 dis.)	Endocrine–metabolic (1.2%; 5 dis.)	Metabolic (1.5%; 4 dis.)	Metabolic (9.7%; 4 dis.)	Metabolic (33.0%; 4 dis.)	Metabolic (23.9%; 5 dis.)	Metabolic (21.5%; 4 dis.)
		Mental health (0.2%; 2 dis.)	Neuromusc. ^c^–depressive (6.4%; 13 dis.)	Neuromusc.–depressive (7.2%; 9 dis.)	Neuromusc.– depressive (18.6%; 8 dis.)	Neuromusc.–degenerative (37.2%; 10 dis.)	Neuromusc.–degenerative (24.6%; 9 dis.)
					Cardiorespiratory (3.4%; 9 dis.)	Cardiorespiratory (12.3%; 13 dis.)	Cardiorespiratory (12.9%; 9 dis.)
**Men**	**0–14**	**15–29**	**30–44**	**45–59**	**60–74**	**75–89**	**≥90**
Number	2	3	4	4	4	4	3
Name (prevalence; No. of dis.)	Growth–dev. (1.0%; 4 dis.)	Allergic–growth–dev. (1.9%; 6 dis.)	Allergic (0.4%; 3 dis.)	Cardiovascular (0.4%; 3 dis.)	Cardiorespiratory (7.0%; 10 dis.)	Cardiorespiratory (19.3%; 16 dis.)	Cardiorespiratory (17.2%; 9 dis.)
	Allergic (2.8%; 2 dis.)	Metabolic (0.1%; 2 dis.)	Metabolic (2.5%; 4 dis.)	Metabolic (14.3%; 5 dis.)	Metabolic (33.3%; 5 dis.)	Metabolic (35.1%; 5 dis.)	Neuromusc.– degenerative (46.9%; 12 dis.)
		Mental health (0.2%; 3 dis.)	Mental health (0.5%; 3 dis.)	Mental health (0.1%; 2 dis.)	Peripheral vascular (0.3%; 2 dis.)	Neurodegenerative–vascular (5.4%; 5 dis.)	Neurodegenerative–vascular (4.8%; 3 dis.)
			Sensory processing (0.1%; 2 dis.)	Neuromusc.– depressive (4.9%; 10 dis.)	Neuromusc.– depressive (11.2%; 8 dis.)	Neuromusc.– degenerative (15.5%; 5 dis.)	

^a^ Number of diseases composing each pattern; ^b^ developmental; ^c^ musculoskeletal.

## Supplementary Materials

The following are available online at https://www.mdpi.com/1660-4601/17/12/4242/s1, Table S1: Results of the exploratory factor analysis in women; Table S2: Results of the exploratory factor analysis in men.

## References

[1] Barnett K., Mercer S.W., Norbury M., Watt G., Wyke S., Guthrie B. (2012). Epidemiology of multimorbidity and implications for health care, research, and medical education: A cross-sectional study. Lancet.

[2] Salisbury C., Johnson L., Purdy S., Valderas J.M., Montgomery A.A. (2011). Epidemiology and impact of multimorbidity in primary care: A retrospective cohort study. Br. J. Gen. Pract..

[3] Palmer K., Marengoni A., Forjaz M.J., Jureviciene E., Laatikainen T., Mammarella F., Muth C., Navickas R., Prados-Torres A., Rijken M. (2018). Multimorbidity care model: Recommendations from the consensus meeting of the Joint Action on Chronic Diseases and Promoting Healthy Ageing across the Life Cycle (JA-CHRODIS). Health Policy.

[4] Palmer K., Carfì A., Angioletti C., Di Paola A., Navickas R., Dambrauskas L., Jureviciene E., João Forjaz M., Rodriguez-Blazquez C., Prados-Torres A. (2019). A Methodological Approach for Implementing an Integrated Multimorbidity Care Model: Results from the Pre-Implementation Stage of Joint Action CHRODIS-PLUS. Int. J. Environ. Res. Public Health.

[5] Muth C., van den Akker M., Blom J.W., Mallen C.D., Rochon J., Schellevis F.G., Becker A., Beyer M., Gensichen J., Kirchner H. (2014). The Ariadne principles: How to handle multimorbidity in primary care consultations. BMC Med..

[6] Muth C., Uhlmann L., Haefeli W.E., Rochon J., van den Akker M., Perera R., Güthlin C., Beyer M., Oswald F., Valderas J.M. (2018). Effectiveness of a complex intervention on Prioritising Multimedication in Multimorbidity (PRIMUM) in primary care: Results of a pragmatic cluster randomised controlled trial. BMJ Open.

[7] Prados-Torres A., del Cura-González I., Prados-Torres D., López-Rodríguez J.A., Leiva-Fernández F., Calderón-Larrañaga A., López-Verde F., Gimeno-Feliu L.A., Escortell-Mayor E., Pico-Soler V. (2017). Effectiveness of an intervention for improving drug prescription in primary care patients with multimorbidity and polypharmacy: Study protocol of a cluster randomized clinical trial (Multi-PAP project). Implement. Sci..

[8] Salisbury C., Man M.-S., Bower P., Guthrie B., Chaplin K., Gaunt D.M., Brookes S., Fitzpatrick B., Gardner C., Hollinghurst S. (2018). Management of multimorbidity using a patient-centred care model: A pragmatic cluster-randomised trial of the 3D approach. Lancet.

[9] McCarthy C., Clyne B., Corrigan D., Boland F., Wallace E., Moriarty F., Fahey T., Hughes C., Gillespie P., Smith S.M. (2017). Supporting prescribing in older people with multimorbidity and significant polypharmacy in primary care (SPPiRE): A cluster randomised controlled trial protocol and pilot. Implement. Sci..

[10] Garvey J., Connolly D., Boland F., Smith S.M. (2015). OPTIMAL, an occupational therapy led self-management support programme for people with multimorbidity in primary care: A randomized controlled trial. BMC Fam. Pract..

[11] Dolovich L., Oliver D., Lamarche L., Thabane L., Valaitis R., Agarwal G., Carr T., Foster G., Griffith L., Javadi D. (2019). Combining volunteers and primary care teamwork to support health goals and needs of older adults: A pragmatic randomized controlled trial. CMAJ.

[12] Prados-Torres A., Poblador-Plou B., Gimeno-Miguel A., Calderón-Larrañaga A., Poncel-Falcó A., Gimeno-Feliú L.A., González-Rubio F., Laguna-Berna C., Marta-Moreno J., Clerencia-Sierra M. (2018). Cohort Profile: The Epidemiology of Chronic Diseases and Multimorbidity. The EpiChron Cohort Study. Int. J. Epidemiol..

[13] Prados-Torres A., Poblador-Plou B., Calderón-Larrañaga A., Gimeno-Feliu L.A., González-Rubio F., Poncel-Falcó A., Sicras-Mainar A., Alcalá-Nalvaiz J.T. (2012). Multimorbidity patterns in primary care: Interactions among chronic diseases using factor analysis. PLoS ONE.

[14] Calderón-Larrañaga A., Gimeno-Feliu L.A., González-Rubio F., Poblador-Plou B., Lairla-San José M., Abad-Díez J.M., Poncel-Falcó A., Prados-Torres A. (2013). Polypharmacy patterns: Unravelling systematic associations between prescribed medications. PLoS ONE.

[15] Poblador-Plou B., Van Den Akker M., Vos R., Calderón-Larrañaga A., Metsemakers J., Prados-Torres A. (2014). Similar multimorbidity patterns in primary care patients from two European regions: Results of a factor analysis. PLoS ONE.

[16] Poblador-Plou B., Calderon-Larranaga A., Marta-Moreno J., Hancco-Saavedra J., Sicras-Mainar A., Soljak M., Prados-Torres A. (2014). Comorbidity of dementia: A cross-sectional study of primary care older patients. BMC Psychiatry.

[17] Clerencia-Sierra M., Calderón-Larrañaga A., Martínez-Velilla N., Vergara-Mitxeltorena I., Aldaz-Herce P., Poblador-Plou B., Machón-Sobrado M., Egüés-Olazabal N., Abellán-Van Kan G., Prados-Torres A. (2015). Multimorbidity patterns in hospitalized older patients: Associations among chronic diseases and geriatric syndromes. PLoS ONE.

[18] Diaz E., Poblador-Pou B., Gimeno-Feliu L.A., Calderón-Larrañaga A., Kumar B.N., Prados-Torres A. (2015). Multimorbidity and its patterns according to immigrant origin. A nationwide register-based study in Norway. PLoS ONE.

[19] Menditto E., Miguel A.G., Juste A.M., Plou B.P., Pascual-Salcedo M.A., Orlando V., Rubio F.G., Torres A.P. (2019). Patterns of multimorbidity and polypharmacy in young and adult population: Systematic associations among chronic diseases and drugs using factor analysis. PLoS ONE.

[20] Gimeno-Miguel A., Gracia Gutiérrez A., Poblador-Plou B., Coscollar-Santaliestra C., Pérez-Calvo J.I., Divo M.J., Calderón-Larrañaga A., Prados-Torres A., Ruiz-Laiglesia F.J. (2019). Multimorbidity patterns in patients with heart failure: An observational Spanish study based on electronic health records. BMJ Open.

[21] (2011). European Commission Toolkit Gender in EU-Funded Research; Luxembourg. https://eige.europa.eu/library/resource/aleph_eige000000269.

[22] The Johns Hopkins University Johns Hopkins ACG® System. https://www.hopkinsacg.org/.

[23] Caughey G.E., Roughead E.E. (2011). Multimorbidity Research Challenges: Where to Go from Here?. J. Comorbidity.

[24] Westert G.P., Satariano W.A., Schellevis F.G., van den Bos G.A. (2001). Patterns of comorbidity and the use of health services in the Dutch population. Eur. J. Public Health.

[25] Navickas R., Petric V.-K., Feigl A.B., Seychell M. (2016). Multimorbidity: What do we know? What should we do?. J. Comorbidity.

[26] Rijken M., Struckmann V., Dyakova M., Melchiorre M.G., Rissanen S., Van Ginneken E. (2013). ICARE4EU: Improving care for people with multiple chronic conditions in Europe. Eurohealth (Lond.).

[27] Mahmood S.S., Levy D., Vasan R.S., Wang T.J. (2014). The Framingham Heart Study and the epidemiology of cardiovascular disease: A historical perspective. Lancet.

[28] Piepoli M.F., Hoes A.W., Agewall S., Albus C., Brotons C., Catapano A.L., Cooney M.T., Corrà U., Cosyns B., Deaton C. (2016). 2016 European guidelines on cardiovascular disease prevention in clinical practice. The Sixth Joint Task Force of the European Society of Cardiology and Other Societies on Cardiovascular Disease Prevention in Clinical Practice (constituted by representatives of 10 societies and by invited experts. Developed with the special contribution of the European Association for Cardiovascular Prevention & Rehabilitation. Eur. Heart J..

[29] Mercer S.W., Guthrie B., Furler J., Watt G.C.M., Hart J.T. (2012). Multimorbidity and the inverse care law in primary care. BMJ.

[30] Marengoni A., Rizzuto D., Wang H.X., Winblad B., Fratiglioni L. (2009). Patterns of chronic multimorbidity in the elderly population. J. Am. Geriatr. Soc..

[31] Ng S.K., Holden L., Sun J. (2012). Identifying comorbidity patterns of health conditions via cluster analysis of pairwise concordance statistics. Stat. Med..

[32] Smith S.M., Wallace E., O’Dowd T., Fortin M. (2016). Interventions for improving outcomes in patients with multimorbidity in primary care and community settings. Cochrane Database Syst. Rev..

[33] Fortin M., Stewart M., Poitras M.E., Almirall J., Maddocks H. (2012). A systematic review of prevalence studies on multimorbidity: Toward a more uniform methodology. Ann. Fam. Med..

[34] Ng S.K., Tawiah R., Sawyer M., Scuffham P. (2018). Patterns of multimorbid health conditions: A systematic review of analytical methods and comparison analysis. Int. J. Epidemiol..

[35] Wilson P.W.F., Kannel W.B., Silbershatz H., D’Agostino R.B. (1999). Clustering of metabolic factors and coronary heart disease. Arch. Intern. Med..

[36] Grundy S.M., Brewer H.B., Cleeman J.I., Smith S.C., Lenfant C., American Heart Association, National Heart, Lung, and Blood Institute (2004). Definition of Metabolic Syndrome: Report of the National Heart, Lung, and Blood Institute/American Heart Association Conference on Scientific Issues Related to Definition. Circulation.

[37] Alberti G., Zimmet P., Shaw J., Grundy S. (2006). The IDF Consensus Worldwide Definition of the Metabolic Syndrome.

[38] Abad-Díez J.M., Calderón-Larrañaga A., Poncel-Falcó A., Poblador-Plou B., Calderón-Meza J.M., Sicras-Mainar A., Clerencia-Sierra M., Prados-Torres A. (2014). Age and gender differences in the prevalence and patterns of multimorbidity in the older population. BMC Geriatr..

[39] Choi H.K., De Vera M.A., Krishnan E. (2008). Gout and the risk of type 2 diabetes among men with a high cardiovascular risk profile. Rheumatology.

[40] Rho Y.H., Choi S.J., Lee Y.H., Ji J.D., Choi K.M., Baik S.H., Chung S., Kim C.-G., Choe J.-Y., Lee S.W. (2005). The Prevalence of Metabolic Syndrome in Patients with Gout: A Multicenter Study. J. Korean Med. Sci..

[41] Schäfer I., von Leitner E.C., Schön G., Koller D., Hansen H., Kolonko T., Kaduszkiewicz H., Wegscheider K., Glaeske G., van den Bussche H. (2010). Multimorbidity patterns in the elderly: A new approach of disease clustering identifies complex interrelations between chronic conditions. PLoS ONE.

[42] Wang R., Yan Z., Liang Y., Tan E.C.K., Cai C., Jiang H., Song A., Qiu C. (2015). Prevalence and patterns of chronic disease pairs and multimorbidity among older Chinese adults living in a rural area. PLoS ONE.

[43] Garin N., Koyanagi A., Chatterji S., Tyrovolas S., Olaya B., Leonardi M., Lara E., Koskinen S., Tobiasz-Adamczyk B., Luis Ayuso-Mateos J. (2016). Global Multimorbidity Patterns: A Cross-Sectional, Population-Based, Multi-Country Study. J. Gerontol. Ser. A Biol. Sci. Med..

[44] Walker V., Perret-Guillaume C., Kesse-Guyot E., Agrinier N., Hercberg S., Galan P., Assmann K.E., Briançon S., Rotonda C. (2016). Effect of multimorbidity on health-related quality of life in adults aged 55 years or older: Results from the SU.VI.MAX 2 Cohort. PLoS ONE.

[45] Morgan A.D., Zakeri R., Quint J.K. (2018). Defining the relationship between COPD and CVD: What are the implications for clinical practice?. Ther. Adv. Respir. Dis..

[46] Carter P., Lagan J., Fortune C., Bhatt D.L., Vestbo J., Niven R., Chaudhuri N., Schelbert E.B., Potluri R., Miller C.A. (2019). Association of Cardiovascular Disease With Respiratory Disease. J. Am. Coll. Cardiol..

[47] Vestbo J., Anderson J.A., Brook R.D., Calverley P.M.A., Celli B.R., Crim C., Martinez F., Yates J., Newby D.E. (2016). SUMMIT Investigators. Fluticasone Furoate and Vilanterol and Survival in Chronic Obstructive Pulmonary Disease With Heightened Cardiovascular Risk (SUMMIT): A Double-Blind Randomised Controlled Trial. Lancet.

[48] Lamers F., Van Oppen P., Comijs H.C., Smit J.H., Spinhoven P., Van Balkom A.J.L.M., Nolen W.A., Zitman F.G., Beekman A.T.F., Penninx B.W.J.H. (2011). Comorbidity patterns of anxiety and depressive disorders in a large cohort study: The Netherlands Study of Depression and Anxiety (NESDA). J. Clin. Psychiatry.

[49] Kirchberger I., Meisinger C., Heier M., Zimmermann A.K., Thorand B., Autenrieth C.S., Peters A., Ladwig K.H., Döring A. (2012). Patterns of multimorbidity in the aged population. results from the KORA-Age study. PLoS ONE.

[50] Gureje O., Von Korff M., Kola L., Demyttenaere K., He Y., Posada-Villa J., Lepine J.P., Angermeyer M.C., Levinson D., de Girolamo G. (2008). The relation between multiple pains and mental disorders: Results from the World Mental Health Surveys. Pain.

[51] Cabrera-León A., Cantero-Braojos M.Á., Garcia-Fernandez L., Guerra De Hoyos J.A. (2018). Living with disabling chronic pain: Results from a face-to-face cross-sectional population-based study. BMJ Open.

[52] Goldenberg D.L. (2010). The Interface of Pain and Mood Disturbances in the Rheumatic Diseases. Semin. Arthritis Rheum..

[53] Chopra K., Arora V. (2014). An intricate relationship between pain and depression: Clinical correlates, coactivation factors and therapeutic targets. Expert Opin. Ther. Targets.

[54] Kiecolt-Glaser J.K., Derry H.M., Fagundes C.P. (2015). Inflammation: Depression Fans the Flames and Feasts on the Heat. Am. J. Psychiatry.

[55] Berk M., Williams L.J., Jacka F.N., O’Neil A., Pasco J.A., Moylan S., Allen N.B., Stuart A.L., Hayley A.C., Byrne M.L. (2013). So depression is an inflammatory disease, but where does the inflammation come from?. BMC Med..

[56] Nerurkar L., Siebert S., McInnes I.B., Cavanagh J. (2019). Rheumatoid arthritis and depression: An inflammatory perspective. Lancet Psychiat..

[57] Krishnadas R., Cavanagh J. (2012). Depression: An inflammatory illness?. J. Neurol. Neurosurg. Psychiatry.

[58] Ciaran P.C., Galts L.E.B., Bettio D.C., Jewett C.C., Yang P.S., Brocardo A.L.S., Rodrigues J.S., Thacker J.G.M. (2019). Depression in neurodegenerative diseases: Common mechanisms and current treatment options. Neurosci. Biobehav. Rev..

[59] Schrag A., Taddei R.N. (2017). Depression and Anxiety in Parkinson’s Disease. Int. Rev. Neurobiol..

[60] Rubin R. (2018). Exploring the Relationship Between Depression and Dementia. JAMA.

[61] Conway K.P., Compton W., Stinson F.S., Grant B.F. (2006). Lifetime comorbidity of DSM-IV mood and anxiety disorders and specific drug use disorders: Results from the National Epidemiologic Survey on Alcohol and Related Conditions. J. Clin. Psychiatry.

[62] Grant B.F., Saha T.D., June Ruan W., Goldstein R.B., Patricia Chou S., Jung J., Zhang H., Smith S.M., Pickering R.P., Huang B. (2016). Epidemiology of DSM-5 drug use disorder results from the national epidemiologic survey on alcohol and related conditions-III. JAMA Psychiatry.

[63] Lai H.M.X., Cleary M., Sitharthan T., Hunt G.E. (2015). Prevalence of comorbid substance use, anxiety and mood disorders in epidemiological surveys, 1990-2014: A systematic review and meta-analysis. Drug Alcohol Depend..

[64] Khokhar J.Y., Dwiel L.L., Henricks A.M., Doucette W.T., Green A.I. (2018). The link between schizophrenia and substance use disorder: A unifying hypothesis. Schizophr. Res..

[65] Holton A.E., Gallagher P., Fahey T., Cousins G. (2017). Concurrent use of alcohol interactive medications and alcohol in older adults: A systematic review of prevalence and associated adverse outcomes. BMC Geriatr..

